# β-Asarone increases doxorubicin sensitivity by suppressing NF-κB signaling and abolishes doxorubicin-induced enrichment of stem-like population by destabilizing Bmi1

**DOI:** 10.1186/s12935-019-0873-3

**Published:** 2019-06-03

**Authors:** Li-Na Lv, Xiao-Chao Wang, Li-Ju Tao, Hong-Wen Li, Shu-You Li, Fei-Meng Zheng

**Affiliations:** 1 Department of Medical Oncology of The Eastern Hospital, The First Affiliated Hospital, Sun Yat-sen University, No. 58, Zhong Shan Er Lu, Guangzhou, 510080 China; 2 Department of Hematology, Wuming Hospital of Guangxi Medical University, Nanning, China; 3 Department of Hematology, Affiliated Hospital of Youjiang Medical University for Nationalities, Baise, China; 4 Guangdong Provincial Key Laboratory of Orthopedics and Traumatology, The First Affiliated Hospital, Sun Yat-sen University, Guangzhou, China; 5 Department of Medical Oncology, Wuming Hospital of Guangxi Medical University, Nanning, China

**Keywords:** β-Asarone, Lymphoma, Natural compounds, Bmi1, Cancer stem cell, NF-κB, Synergistic cytotoxic effects, Doxorubicin

## Abstract

**Background:**

Lymphoma is one of the most common hematologic malignancy. Drug resistance is the main obstacle faced in lymphoma treatment. Cancer stem cells are considered as the source of tumor recurrence, metastasis and drug resistance. The β-Asarone, a low-toxicity compound from the traditional medical herb *Acorus calamus*, has been shown to act as an anti-cancer reagent in various cancer types. However, the anti-cancer activities of β-Asarone in lymphoma have not been shown.

**Methods:**

Cell counting assay was used to evaluate Raji cell proliferation. CCK8 assay was used to evaluate the cell viability. Annexin-V/PI staining and flow cytometry analysis were used to evaluate apoptosis. ALDEFLUOR assay was used to evaluate the stem-like population. Luciferase reporter assay was used to examine the activation of NF-κB signaling. Western blot and polymerase chain reaction (PCR) were used to determine the expression of interested genes.

**Results:**

We showed that β-Asarone inhibited proliferation and induced apoptosis in Raji lymphoma cells in a dose-dependent manner. Additionally, β-Asarone functioned as a sensitizer of doxorubicin and resulted in synergistic effects on inhibition of proliferation and induction of apoptosis when combined with doxorubicin treatment. Interestingly, we found that β-Asarone also reduced the stem-like population of Raji lymphoma cells in a dose-dependent manner, and suppressed the expression of c-Myc and Bmi1. Importantly, β-Asarone abolished doxorubicin-induced enrichment of the stem-like population. In the mechanism study, we revealed that β-Asarone suppressed not only basal NF-κB activity but also Tumor necrosis factor α (TNF-α) induced NF-κB activity. Moreover, blocking NF-κB signaling inactivation was critical for β-Asarone induced apoptosis and inhibition of proliferation, but not for the effect on β-Asarone reduced stem-like population. In fact, β-Asarone suppressed stem-like population by destabilizing Bmi1 via a proteasome-mediated mechanism.

**Conclusions:**

Our data suggested the application of β-Asarone to lower the toxic effect of doxorubicin and increase the sensitivity of doxorubicin in clinical treatment. More importantly, our data revealed a novel role of β-Asarone which could be used to eliminate stem-like population in lymphoma, implying that β-Asarone might reduce relapse and drug resistance.

**Electronic supplementary material:**

The online version of this article (10.1186/s12935-019-0873-3) contains supplementary material, which is available to authorized users.

## Background

Lymphoma is one of the most common hematologic malignancy. Treatment for lymphoma may involve one or more of the following strategies: chemotherapy, radiation therapy, targeted therapy, and immunotherapy. Doxorubicin is a commonly used and effective chemotherapy drug in the first-line chemotherapy regimens. However, the application of doxorubicin is limited because of adverse effects and drug resistance [1]. Thus, the development of novel anti-cancer drugs with few toxic effects and the sensitizing effect is one of the main focuses in lymphoma research.

Cancer stem cells are a small population of cells within the tumor with the abilities for self-renewal, differentiation, and tumorigenicity [2]. The existing of cancer stem cells are thought to be the major obstacle for cancer treatment due to their considerable chemo- and radio-resistance. Cancer stem cells are considered as the source of tumor recurrence and metastasis [2]. Thus, the development of drug targeting cancer stem cells becomes essential in treating cancer and preventing tumor relapse.

Natural products are usually the sources for developing novel drug with high efficacy and few side effects for treating diseases. Currently, a significant number of drugs with different mechanisms that are used to treat cancer preclinically are derived from natural products. For example, bitter melon extract displayed anti-cancer activities in multiple cancers by decreasing the infiltrating regulatory T cells and Th17 cells in the tumor [3], enhancing natural killer-mediated toxicity [4] and inducing autophagic cell death [5]. Berberine functions as a strong anti-cancer compound by inducing apoptosis and cell cycle arrest at the G2/M phase [6]. Flavonoids play a critical role in the treatment of a large number of cancers by inhibiting DNA topoisomerase I [7] and cyclooxygenase [8].

The β-Asarone (1-propenyl-2,4,5-methoxybenzol) is one of the main bioactive constituents of the traditional medical herb Acorus calamus [9]. Numerous studies have illustrated that β-Asarone displays multiple activities such as anti-inflammatory [10], anti-fungal [11], anti-epileptic [12] and anti-depressant [13] activities. Currently, growing evidence has revealed that β-Asarone functions as an anti-cancer reagent in colorectal cancer [14], gastric cancer [15], and lung cancer [16]. However, there is no information about the anti-cancer activities of β-Asarone in lymphomas, and the underlying molecular mechanisms are largely unknown. Besides, whether β-Asarone might display activity against cancer stem cell has not been explored.

NF-κB family consists of five members, c-rel, p65, Rel B, p50/p105, and p52/p100, which form various hetero- or homodimers [17]. During activation, IκBα protein is phosphorylated and degraded by the proteasome. The proteolysis of IκBα allows NF-κB to translocate into the nucleus and subsequently activate the expression of multiple downstream genes involved in cell growth, differentiation, and survival [18]. NF-κB signaling is a critical pathway involved in immune and inflammatory cellular responses affecting both cell growth, differentiation and survival, and is deregulated in multiple human diseases including chronic inflammatory diseases and cancers [18]. Various subtypes of human lymphoma rely on the constitutive activity of the NF-κB pathway for survival [19]. Thus, the development of inhibitors targeted NF-κB signaling is an attractive strategy to treat many inflammatory diseases and cancers, including lymphomas. Currently, little is known about whether β-Asarone might produce anti-cancer effects in lymphomas through interfering NF-κB signaling activation.

Bmi1 is a regulatory component of epigenetic repressor polycomb group proteins [20]. It plays a critical role in the repression of gene transcription via chromatin modulation and modification of histones [21]. Bmi1 participates in the regulation of multiple cellular processes during development, including cell cycle progression, senescence, aging, apoptosis, and angiogenesis [21]. Bmi1 also plays an essential role in the regulation of endogenous stem cells and cancer stem cells function [22]. Whether β-Asarone might participate in the regulation of Bmi1 expression is still unknown.

In this study, we explored the anti-cancer effects of β-Asarone in lymphoma cells and revealed a potential molecular mechanism. We showed that β-Asarone functioned as a sensitizer to induce synergistic effects on proliferation and apoptosis when combined with doxorubicin treatment by blocking NF-κB signaling inactivation. More importantly, we revealed a novel function of β-Asarone which acted as an inhibitor of the stem-like population in lymphoma cells by destabilizing Bmi1 via a proteasome-mediated mechanism. Thus, our data implicated that β-Asarone could be a promising anti-cancer reagent by suppressing NF-κB activity and Bmi1 expression in lymphoma. Our data also provided a strategy to target stem-like population, lower drug dose and increase drug sensitivity of doxorubicin.

## Materials and methods

### Reagents and cell culture

β-Asarone, doxorubicin, JSH-23, and Annexin V-FITC Apoptosis Detection Kit were purchased from Sigma-Aldrich. Antibodies against NF-κB/p65, p-NF-κB (Ser536), Lamin A, IκBα, octamer-binding transcription factor 4 (Oct4), SRY (sex determining region Y)-box 2 (Sox2), Kruppel-like factor 4 (Klf4), c-Myc, Bmi1, Caspase-3, Caspase-8, Caspase-9, poly ADP ribose polymerase (PARP), and glyceraldehyde 3-phosphate dehydrogenase (GAPDH) were purchased from Cell Signaling Technology. β-Asarone was dissolved in dimethylsulfoxide (DMSO) to a stock concentration of 500 mM and stored at − 20 °C. Raji cells were obtained from the American Type Culture Collection (ATCC). Raji cells were cultured in RPMI-1640 (Gibco) supplemented with 10% fetal bovine serum (Gibco). Cells were maintained at 37 °C in a humidified 5% CO_2_ atmosphere.

### Cell counting assay

2 ml (1 × 10^4^/ml) cells were plated per well of a 6 well plate. Cells were treated with drug for indicated concentration and time. 50 μl of cells was mixed with 50 μl of 0.4% trypan blue by gently pipetting. 20 μl of the mix was loaded into each chamber of the hemocytometer. Counting were performed by triplicate by one analyst under a 40× objective.

### CCK8 assay

Viable cells were measured using CCK8 assay (Dojindo). Briefly, cells were plated into 96-well plates at a density of 1 × 10^3^ cells per well and incubated overnight in a 5% CO_2_ atmosphere at 37 °C before exposure to drugs. Then, cells were treated with or without the indicated concentrations of drugs for the indicated time. Subsequently, CCK8 reagents were added to each well and cells were incubated for another 1 h at 37 °C. The absorbance of optical density at 450 nm was determined using Micro-plate Reader (Bio-Rad) at 450 nm.

### Annexin V/PI analysis

Cells were treated with the indicated concentration of β-Asarone or doxorubicin. Cells were collected and resuspended in binding buffer (500 μl/sample). Cells were stained with Annexin-V-FITC (5 μl/sample) followed by propidium iodide (PI) (5 μl/sample). Cells were incubated for 15 min in the dark at 4 °C and subjected to flow cytometry (BD Biosciences).

### Sphere formation assay

The sphere formation assay and the sphere passage assay were performed as previously described [23, 24]. Single cells were plated in ultralow attachment 6-well plates at a density of 500 viable cells/ml. The cells were maintained in sphere culture medium, DMEM/F12 (Gibco) supplemented with B27 (Thermo Fisher Scientific), 20 ng/ml epidermal growth factor (EGF) (Sigma-Aldrich), 20 ng/ml basic fibroblast growth factor (bFGF) (BD Biosciences) and 4 μg/ml heparin (Sigma-Aldrich) for 3–6 days. The spheres were photographed using an inverted microscope (100×, Olympus). The diameters of the spheres were calculated with the Image pro plus 6.0 software (Media Cybernetics). For sphere passage assay, spheres were repeatedly pipetted to detach cells from spheres. Clumped cells were excluded with a 40 μm sieve. Then, sphere formation assay was performed.

### Combination index (CI) calculation

CI was analyzed with the CompuSyn software using the average fraction of cells that responded to each drug [25]. CI values of less than 0.9, between 0.9 and 1.1 and more than 1.1 were defined as synergistic, additive and antagonistic, respectively.

### Intracellular influx of doxorubicin

Intracellular influx of doxorubicin was performed as previously described [26]. Cells were treated with or without β-Asarone or doxorubicin for the indicated time. Cells were harvested and resuspended in phosphate buffered saline. The excitation and emission wavelengths used for doxorubicin was 488 nm, 575/25 nm. Minimum of 10,000 events from each sample was analyzed to generate histograms for the fluorescence intensity.

### Nuclear protein extraction

Cells were treated with Buffer I (25 mM HEPES pH 7.9, 5 mM KCl, 0.5 mM MgCl_2_, and 1 mM DTT) for 5 min. Then, an equal volume of Buffer II (25 mM HEPES pH 7.9, 5 mM KCl, 0.5 mM MgCl_2_, 1 mM DTT and 0.4% (v/v) NP40 supplemented with protease and phosphatase inhibitors) was added. Samples were incubated with rotation at 4 °C for 15 min. The lysates were centrifuged for 5 min at 4 °C at 2500 rpm. To obtain nuclear extracts, the pellets were treated with Buffer III (25 mM HEPES, pH 7.9, 400 mM NaCl, 10% sucrose or dextrose, 0.05% NP-40 and 1 mM DTT supplemented with protease and phosphatase inhibitors). Pellets were incubated for 1 h at 4 °C, the lysates were centrifuged, for 10 min, at 4 °C at 10,000*g*. Supernatants after this spin contained the nuclear protein preparation [24].

### Western blot analysis

Cells treated with the indicated concentration of β-Asarone or TNF-α for an indicated time were lysed in RIPA buffer. The protein concentration was determined by Bradford method. Equal amounts of cell extracts were subjected to electrophoresis in SDS-polyacrylamide gels and transferred to nitrocellulose membrane (Millipore). Nitrocellulose membranes were blocked by 3% BSA in TBST at room temperature (RT). Nitrocellulose membranes were then incubated with different antibodies at 4 °C overnight, followed by incubation for 1 h at room temperature with the appropriate secondary antibodies. Antibody binding was detected with an enhanced chemiluminescence kit (Pierce).

### Semi-quantitative PCR

cDNA was synthesized using the First Strand cDNA synthesis kit (Sigma-Aldrich) according to the manufacturer’s instructions. PCR was performed by using Premix Taq™ (TAKARA) according to the manufacturer’s instructions. Primers used for the Bmi1 gene were 5′-GAGACCAGCAAGTATTGTCC and 5′-TCTTCATCTGCAACCTCTCC. Primers used for the p16INK4a gene were 5′-GAAGGTCCCTCAGACATCCCC and 5′-CCCTGTAGGACCTTCGGTGAC. Primers used for the p14ARF gene were 5′-GTTTTCGTGGTTCACATCCC and 5′-ACCAGCGTGTCCAGGAAG. The GAPDH gene was used as an internal control (primers: 5′-TGCCAAATATGATGACATCAAGAA and 5′-GGAGTGGGTGTCGCTGTTG). PCR was performed using an initial denaturation at 94 °C for 5 min, followed by 18–25 (GAPDH: 18 cycles, Bmi1: 22 cycles, p16INK4a and p14ARF: 25 cycles) cycles at 94 °C for 30 s, 58 °C for 30 s, and 72 °C for 45 s. After amplification, an additional elongation step was performed at 72 °C for 10 min. Amplified PCR products were run on 1.5% agarose gels containing 0.5 g/ml ethidium bromide.

### Luciferase reporter assay

Cells were transiently transfected with NanoLuc Reporter Vector with NF-κB Response Element (Promega), control reporter and/or Renilla luciferase reporter, respectively. After transfection, cells were incubated in medium with the indicated agents. Cells were collected and lysed with lysis buffer. Luciferase activity was determined by using the Dual-Luciferase Assay System according to the manufacturer’s protocol.

### Vector

The shRNA sequences against IκBα (5′-CCGGATCACCAACCAGCCAGAAATTCTCGAGAATTTCTGGCTGGTTGGTGATTTTTT) were cloned into pLVTHM (Addgene). The pLVX-DsRed-Monomer-N1-Bmi-1 vector was constructed by inserting Bmi-1 cDNA containing stop codon into the *Xho*I and *Bam*HI sites of the pLVX-DsRed-Monomer-N1 vector (Clontech).

### Lentivirus preparation and transfection

5 × 10^6^ 293T cells were transfected with 12 μg lentiviral vector, 9 μg psPAX2 and 3 μg pMD2.G. Supernatants were collected every 24 h between 24 and 72 h after transfection, pulled together and concentrated via ultracentrifugation, and the viral titer was determined by serial dilutions. The multiplicity of infection during transfection was 10.

### ALDEFLUOR assay

The ALDH activity was determined by the ALDEFLUOR assay kit (Stem Cell Technologies) according to the manufacturer’s instructions. Cells were collected and incubated with the ALDEFLUOR reagent, with and without specific Aldehyde dehydrogenases (ALDH) inhibitor *N*,*N*-diethylaminobenzaldehyde (DEAB) at 37 °C for 45 min. All stained cells were then examined by using flow cytometry (BD Biosciences).

### Statistical analysis

Statistical analyses were performed using the SPSS software, version 16.0 (SPSS Inc.) or with GraphPad Prism 6.0 (GraphPad Software, Inc.). The ANOVA test, followed by Least Significant Difference test, was used when performing multiple comparisons. The level of significance was set at p < 0.05. The Kruskal–Wallis test, followed by Dunn’s Multiple Comparison test, was used to perform a statistical comparison with regard to spheres size distribution. The level of significance was set at p < 0.05.

## Results

### β-Asarone inhibits proliferation and induces apoptosis in lymphoma cells

**Fig. 1:**
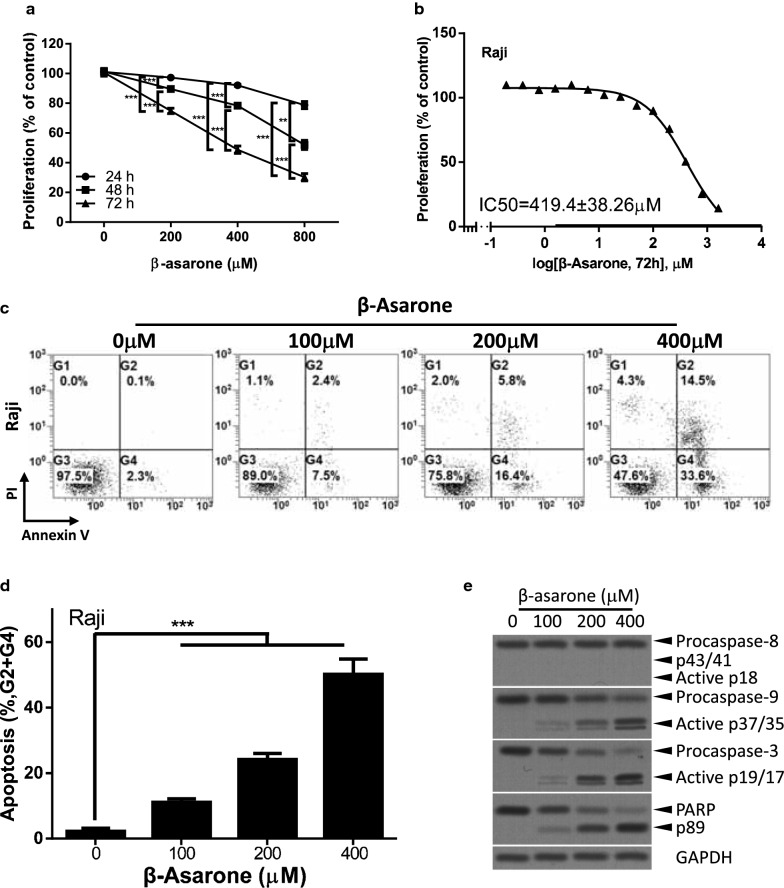
β-Asarone inhibits proliferation and induces apoptosis. **a** Raji cells were treated with the indicated concentration of β-Asarone, cells counting assay was performed at the indicated time points. **b** Cells were treated with various concentration (from 1600 to 0.20 μM, each concentration was 1/2 of the previous one) of β-Asarone for 72 h. The proliferation was examined by cells counting assay. The IC50 were calculated by GraphPad Prism 6.0. **c**–**e** Raji cells were treated with the indicated concentration of β-Asarone for 72 h. Apoptosis was determined by the Annexin V-FITC/PI staining and flow cytometry analysis. Representative results were shown in **c** and statistical results were shown in **d**. The expression of apoptotic proteins was determined by western blot (**e**). The bar represents mean ± SD of three independent experiments (*p < 0.05, **p < 0.01, ***p < 0.001, the ANOVA test, followed by Least Significant Difference test, were used to make statistical comparisons)

We first examined whether β-Asarone suppress proliferation in lymphoma cells. Raji lymphoma cells were treated with various concentrations (0 μM, 200 μM, 400 μM, and 800 μM) of β-Asarone, and cells counting assay was performed at indicated time points (24 h, 48 h, and 72 h) to evaluate cell proliferation. We found that β-Asarone suppressed proliferation of Raji lymphoma cells in dose-dependent and time-dependent manners (Fig. 1a). The IC50 of β-Asarone inhibiting proliferation was 419.4 ± 38.26 μM (Fig. 1b). We next evaluated whether β-Asarone induces apoptosis in lymphoma cells. We treated Raji lymphoma cells with β-Asarone at various concentrations (0 μM, 100 μM, 200 μM, and 400 μM) for 72 h, and the apoptotic cells were evaluated by Annexin V/PI staining and flow cytometry analysis. As shown in Fig. 1c, d, β-Asarone indeed induced apoptosis in a dose-dependent manner in Raji lymphoma cells. Approximately 2.23 ± 0.78% of control cells were Annexin-V positive after control treatment, whereas 11.1 ± 0.88%, 24.17 ± 1.55% or 50.27 ± 3.79% of cells were apoptotic after 72 h of treatment with 100 μM, 200 μM or 400 μM of β-Asarone, respectively (Fig. 1c, d). The expression of apoptotic proteins was also evaluated. β-Asarone mainly induced activation of the intrinsic apoptotic pathway (Fig. 1e). These data indicated that β-Asarone might function as an anti-cancer reagent in lymphoma.

### β-Asarone suppresses stem-like properties

**Fig. 2:**
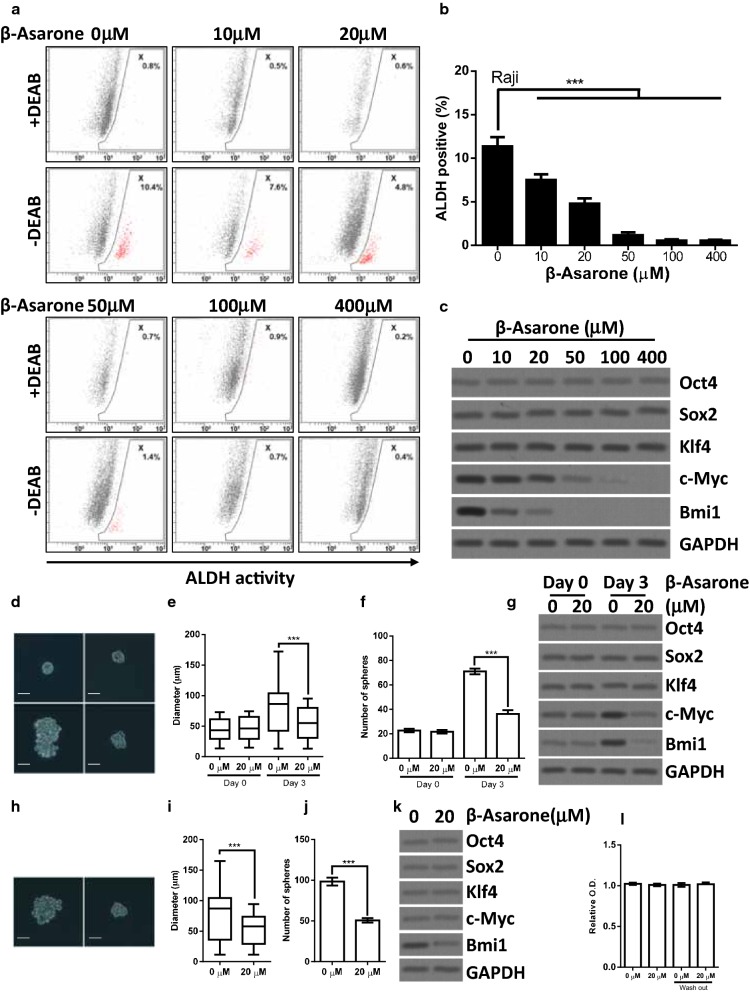
β-Asarone suppresses stem-like properties. **a**, **b** Raji cells were treated with the indicated concentration of β-Asarone for 72 h. ALDH activity was determined by ALDEFLUOR assay and flow cytometry analysis. Representative results were shown in **a** and statistical results were shown in **b**. **c** Raji cells were treated with the indicated concentration of β-Asarone for 72 h. Cells were subjected to western blot analysis. **d**–**g** Raji cells were grown under sphere culture condition for 3 days. Then, the spheres were divided equally into two parts. One was treated with β-Asarone (20 μM) for another 3 days. The other was treated with a control solvent for another 3 days. The spheres were photographed and the diameters of the spheres were calculated. The representative images were shown in **d**. The size distribution of spheres was shown in **e**. Spheres with a diameter larger than 60 μm were counted and shown in **f**. The expression of cancer stem cell-related proteins was determined by western blot (**g**). **h**–**k** β-Asarone was washed out from the sphere. Raji cells were detached from sphere to perform sphere passage assay. The representative images were shown in **h**. The size distribution of spheres was shown in **i**. Spheres with a diameter larger than 60 μm were shown in **j**. The expression of cancer stem cell-related proteins was determined by western blot (**k**). **l** The Raji cells used to perform sphere formation assay and sphere passage assay were also grown under its regular culture medium for 3 days to evaluate proliferation using CCK8 assay. The bar represents mean ± SD of three independent experiments (*p < 0.05, **p < 0.01, ***p < 0.001, the ANOVA test, followed by Least Significant Difference test, were used to make statistical comparisons). The horizontal line within each box in **e** and **i** represented the median value. The Kruskal–Wallis test, followed by Dunn’s Multiple Comparison test, were used to make statistical comparisons (***p < 0.001)

Due to the critical role of cancer stem cells in promoting drug resistance and relapse, we tested whether β-Asarone might act on the stem-like population of Raji lymphoma cells. ALDH is important for the maintenance and differentiation of stem cells and is applied as a biomarker for identifying cancer stem cells population [27]. Raji lymphoma cells were treated with various concentrations (0 μM, 10 μM, 20 μM, 50 μM, 100 μM, and 400 μM) of β-Asarone, and ALDEFLUOR assay was performed to examine the stem-like population. We found that β-Asarone decreased the stem-like population in a dose-dependent manner (Fig. 2a, b). Interestingly, we noticed that the concentration required for β-Asarone to suppress stem-like population was significantly lower than that to inhibit proliferation and induce apoptosis. Moreover, β-Asarone also suppressed the expression of cancer stem cell-related proteins (c-Myc and Bmi1) in a dose-dependent manner (Fig. 2c).

To further confirm the effects of β-Asarone on the stem-like population, we evaluated the stem-like properties under sphere culture condition. Cells were allowed to form spheres for 3 days, and spheres were treated with β-Asarone (20 μM) for another 3 days. As shown in Fig. 2d–f, β-Asarone (20 μM) treatment significantly reduced sphere size and sphere number (Φ ≥ 60 μm). Consistently, the expression of Bmi1 and c-Myc protein was upregulated during the process of sphere formation, while β-Asarone (20 μM) treatment abolished the upregulation of Bmi1 and c-Myc protein (Fig. 2g). We further washed out β-Asarone and performed sphere passage assay using single cell suspension derived from the above sphere culture experiment. Although β-Asarone was washed out, the detrimental effects on sphere formation induced by β-Asarone were still persisted (Fig. 2h–j), leading to inhibition of secondary sphere formation. Importantly, the expression of Bmi1 protein also failed to be recovered upon β-Asarone withdrawing (Fig. 2k). Interestingly, β-Asarone treatment did not cause significant proliferation inhibition under a conventional culture condition (Fig. 2l), indicating that the reduction of sphere formation caused by β-Asarone was induced by inhibition of stem-like properties.

### Synergistic effects of β-Asarone and doxorubicin in lymphoma cells

**Fig. 3:**
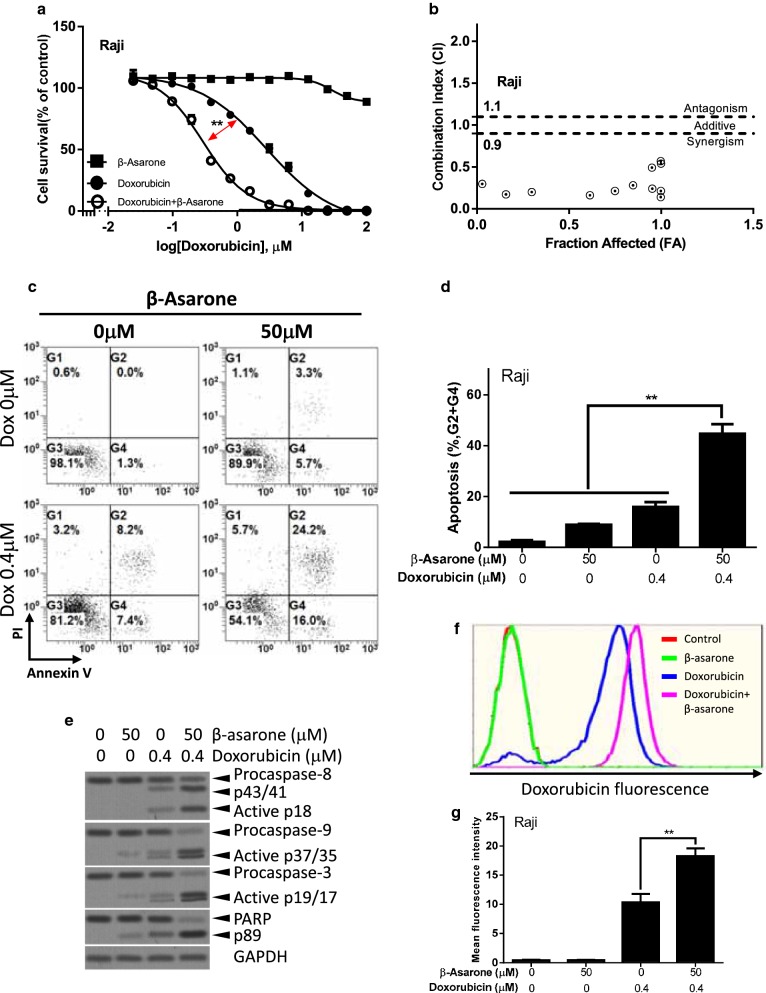
Synergistic effects of β-Asarone and doxorubicin in Raji lymphoma cells. **a** Raji cells were treated with various concentrations of drugs alone or in combination for 72 h. β-Asarone and doxorubicin were combined in a fixed ratio (1:1). The highest concentrations of either drug was 100 μM, the lower concentrations of either drug were 1/2 of previous higher concentrations of either drug. The growth inhibitory effects were determined by an CCK8 assay. **b** The combination indices (CI) were calculated from data obtained in **a**. The plots showed the fraction of Raji cells that were affected by the β-Asarone and doxorubicin combinations. **c**–**g** Raji cells were treated with the indicated concentration of β-Asarone or (and) doxorubicin for 72 h. Apoptosis was evaluated by the Annexin V-FITC/PI staining and flow cytometry analysis. Representative results were shown in **c** and statistical results were shown in **d**. The expression of apoptotic proteins was determined by western blot (**e**). The representative flow cytometric histogram (**f**) of intracellular uptake of doxorubicin and the statistic result were shown in **g**. Bar represents mean ± SD of three independent experiments (*p < 0.05, **p < 0.01, ***p < 0.001, the ANOVA test, followed by Least Significant Difference test, were used to make statistical comparisons)

Doxorubicin is one of the most commonly used and efficacious chemotherapeutic reagents in chemotherapy regimens of lymphoma. We next evaluated whether β-Asarone might affect the cytotoxic effects of doxorubicin. The synergistic analysis was performed to evaluate the interactions between β-Asarone and doxorubicin in Raji lymphoma cells. β-Asarone and doxorubicin were combined in a fixed ratio (1:1). As shown in Fig. 3a, the combination (IC50 = 0.295 ± 0.03 μM, *p *= 0.00127) of β-Asarone and doxorubicin resulted in a greater growth inhibition in Raji lymphoma cells than was achieved with doxorubicin alone (IC50 = 2.843 ± 0.44 μM). To confirm this, we calculated the CI [25]. As shown in Fig. 3b, β-Asarone and doxorubicin indeed acted synergistically to inhibit Raji lymphoma cell proliferation. We further examined the combinational effect of β-Asarone and doxorubicin on apoptosis. Our data showed that the combination (44.47 ± 3.13%) of β-Asarone and doxorubicin resulted in greater apoptosis in Raji lymphoma cells than was achieved with either β-Asarone (8.67 ± 0.29%, *p *= 0.004) or doxorubicin alone (15.63 ± 1.59%, *p *= 0.009) (Fig. 3c, d). Consistently, the synergistic effect of β-Asarone and doxorubicin on the expression of intrinsic apoptotic pathway proteins was also observed (Fig. 3e). Interestingly, the activation of caspase-8, an extrinsic apoptotic pathway marker, induced by doxorubicin were also enhanced by β-Asarone (Fig. 3e). We further examined the effect of β-Asarone on intracellular uptake of doxorubicin. Our data showed that the cellular uptake of doxorubicin was significantly enhanced by β-Asarone treatment (Fig. 3f, g). These data indicated that β-Asarone could be a potential reagent to enhance the cytotoxic effects of doxorubicin.

### β-Asarone abolishes doxorubicin-induced enrichment of stem-like population

**Fig. 4:**
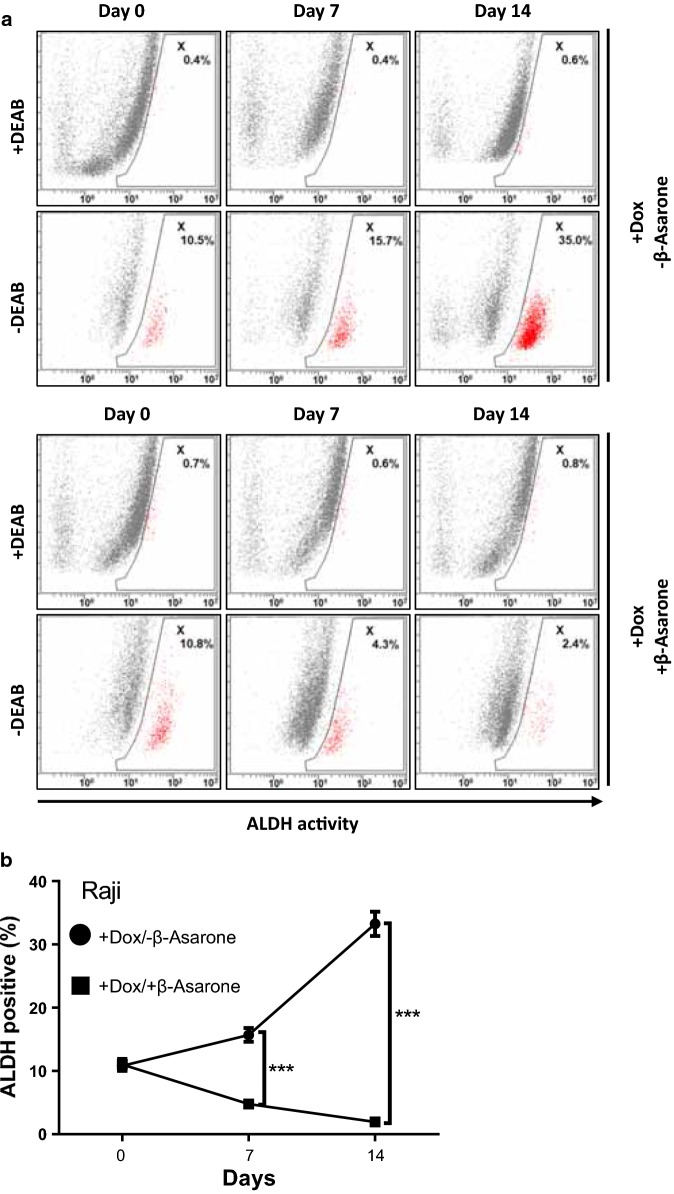
β-Asarone abolishes doxorubicin-induced enrichment of stem-like population. **a**, **b** Raji cells were treated with or without doxorubicin (2 μM) or β-Asarone (20 μM) for the indicated time. ALDH activity was determined by ALDEFLUOR assay and flow cytometry analysis. Representative results were shown in **a** and statistical results were shown in **b**. The bar represents mean ± SD of three independent experiments (*p < 0.05, **p < 0.01, ***p < 0.001, the ANOVA test, followed by Least Significant Difference test, were used to make statistical comparisons)

Chemotherapy preferentially targets the rapidly dividing population of differentiated cells in tumors and spares the slowly growing and chemo-resistant cancer stem cells [2]. Indeed, our data showed that long term treatment of Raji lymphoma cells with doxorubicin resulted in an enrichment of stem-like population, with the percentages at day 0, 7 and 14 were 10.8 ± 0.65%, 15.7 ± 0.90%, and 33.3 ± 1.57%, respectively (Fig. 4a, b). Interestingly, the effect of doxorubicin enriched stem-like population was abolished when combined with β-Asarone treatment. The stem-like population at day 0, 7 and 14 were 11.0 ± 0.75%, 4.77 ± 0.37% and 1.97 ± 0.31%, respectively (Fig. 4a, b). These data suggested a potential application of β-Asarone to overcome doxorubicin resistance.

### β-Asarone inhibits both basal or inducible NF-κB activity

**Fig. 5:**
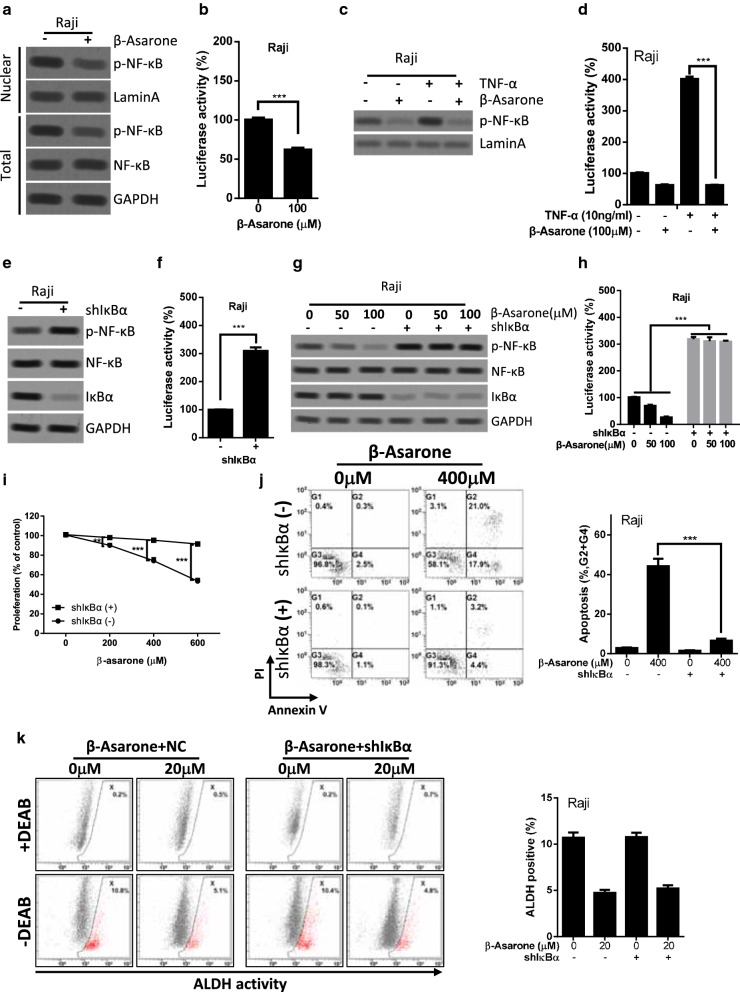
Inhibition of NF-κB activity is critical for β-Asarone induced proliferation inhibition and apoptosis. **a** Raji cells were treated with 100 μM β-Asarone for 72 h. Cells were subjected to nuclear protein or total protein extraction, then western blot analysis was performed. **b** The treatment was the same as **a**. Cells were collected and perform the luciferase reporter assay. **c**, **d** Raji cells were treated with or without 100 μM β-Asarone and (or) 10 ng/ml TNF-α for 72 h. Cells were subjected to nuclear protein extraction and western blot analysis (**c**) or luciferase reporter assay (**d**) (*p < 0.05, **p < 0.01, ***p < 0.001, the ANOVA test, followed by Least Significant Difference test, were used to make statistical comparisons). **e**, **f** Raji cells were expressed IκBα shRNA via lentivirus-mediated gene knockdown. Cells were collected for western blot analysis (**e**) or luciferase reporter assay (**f**). **g**, **h** Raji cells were expressed IκBα shRNA via lentivirus-mediated gene knockdown. Cells were treated with or without the indicated concentration of β-Asarone. Cells were collected for western blot analysis (**g**) or luciferase reporter assay (**h**). **i** Raji cells expressed IκBα shRNA via lentivirus-mediated gene knockdown. IκBα shRNA expressed cells and control cells were treated with or without the indicated concentration of β-Asarone. Cell counting assay was performed at the indicated time points. Bars represent mean ± SD of three independent experiments. **j** Apoptosis was evaluated by the Annexin V-FITC/PI staining and flow cytometry analysis. Representative results were shown in the left panel and statistical results were shown in the right panel. The bar represents mean ± SD of three independent experiments (*p < 0.05, **p < 0.01, ***p < 0.001, the ANOVA test, followed by Least Significant Difference test, were used to make statistical comparisons). **k** Raji cells were expressed IκBα shRNA via lentivirus-mediated gene knockdown. IκBα shRNA expressed cells and control cells were treated with or without the indicated concentration of β-Asarone. ALDEFLUOR assay was performed at the indicated time points. Bars represent mean ± SD of three independent experiments

We next elucidated the mechanism of β-Asarone induced anti-cancer effects in lymphoma cells. NF-κB signaling pathway plays a key role in the survival and proliferation of various human tumors, including lymphoma [19]. The previous study has reported that β-Asarone attenuated pro-inflammatory mediators by blocking IκB degradation to inhibit NF-κB signaling in microglial cells [10]. We, therefore, evaluated whether the anti-cancer effects induced by β-Asarone in lymphoma cells might be caused by inhibition of NF-κB signaling activity. Treatment with 100 μM β-Asarone induced a remarkable decrease in nuclear expression level of NF-κB/p65 with concomitant suppression of NF-κB/p65 phosphorylation at Ser356 in Raji lymphoma cells (Fig. 5a and Additional file 1: Figure S1A). Furthermore, the transcription activity of NF-κB/p65 was significantly reduced in Raji lymphoma cells after 100 μM β-Asarone treatment as determined by luciferase reporter assay (Fig. 5b). Tumor necrosis factor (TNF) is one of the most potent physiological inducers of NF-κB/p65 activation [28]. We investigated whether β-Asarone might suppress TNF-α induced NF-κB activation. As shown in Fig. 5c, TNF-α indeed induced the nuclear translocation of NF-κB/p65, while treatment with β-Asarone abolished TNF-α induced NF-κB/p65 nuclear translocation, and further reduced the basal nuclear expression of NF-κB/p65. Consistently, β-Asarone also abolished TNF-α enhanced NF-κB/p65 transcription activity (Fig. 5d).

### Inhibition of NF-κB activity is critical for β-Asarone induced proliferation inhibition and apoptosis, but not for the β-Asarone reduced stem-like population

We next determined whether inhibition of NF-κB/p65 activity was critical for β-Asarone induced anti-cancer effects. Phosphorylation of IκBα by the IKK complex triggers IκBα polyubiquitination and subsequent degradation by the proteasome, which liberates NF-κB/p65 to translocate into nuclear and activate downstream target genes expression [29]. IκBα expression was downregulated by shRNA to maintain NF-κB/p65 activation. As shown in Fig. 5e, downregulation of IκBα expression by shRNA induced a significant increase of NF-κB/p65 phosphorylation at Ser356 in Raji lymphoma cells. Consistently, the transcription activity of NF‐κB/p65 was significantly enhanced (Fig. 5f). We further observed whether β-Asarone still suppressed NF‐κB/p65 activation when IκBα expression was downregulated by shRNA. As shown in Fig. 5g, β-Asarone failed to suppress NF-κB/p65 activation when IκBα expression was downregulated by shRNA. Accordingly, luciferase reporter assay also showed similar results (Fig. 5h). We next evaluated whether β-Asarone induced apoptosis and inhibition of proliferation depended on inhibition of NF-κB/p65 activation. Our data showed that β-Asarone also failed to induced proliferation inhibition (Fig. 5i) and apoptosis (Fig. 5j) when IκBα expression was suppressed. We further combined β-Asarone and NF-κB/p65 inhibitor JSH-23 [30] treatment to inhibit NF-κB/p65 activation and observe the effect on apoptosis. The combination of these two drugs induced greater apoptosis induction than either drug alone (Additional file 1: Figure S1B). Importantly, we found that downregulation of IκBα expression to maintain NF-κB/p65 activation failed to rescue β-Asarone reduced stem-like population (Fig. 5k). These data implicated that there was others mechanism responsible for the β-Asarone reduced stem-like population.

### Inhibition of Bmi1 expression is critical for the β-Asarone reduced stem-like population

**Fig. 6:**
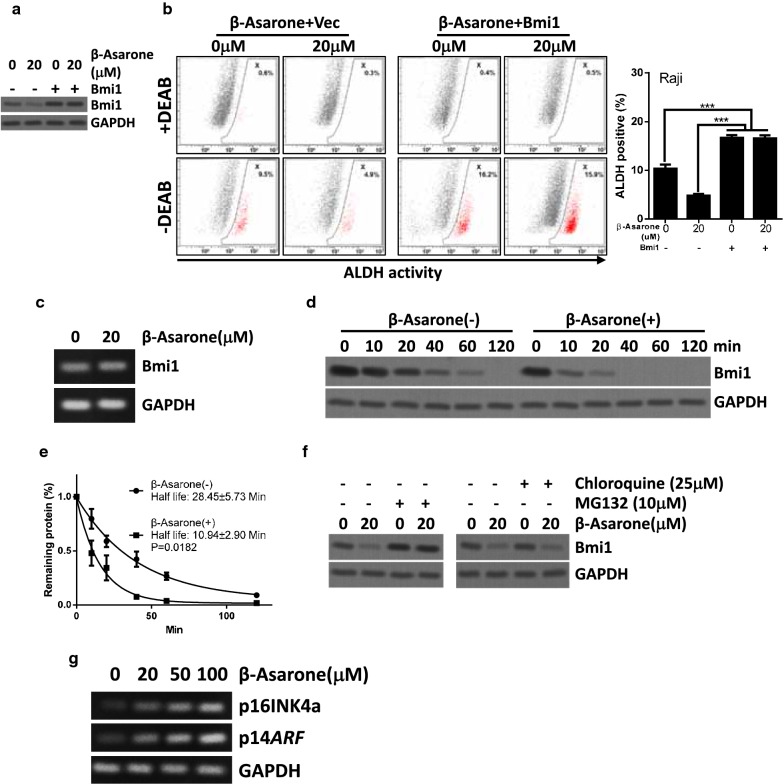
β-Asarone promotes proteasomal degradation of Bmi1 to reduce the stem-like population. **a**, **b** Bmi1 was expressed in Raji cells via lentivirus vector. Cells expressed Bmi1 and control cells were treated with or without the indicated concentration of β-Asarone. Western blot (**a**) or ALDEFLUOR assay (**b**) was performed at the indicated time points. Bars represent mean ± SD of three independent experiments (*p < 0.05, **p < 0.01, ***p < 0.001, the ANOVA test, followed by Least Significant Difference test, were used to make statistical comparisons). **c** Raji cells were treated with or without the indicated concentration of β-Asarone. PCR examined Bim1 and GAPDH expression. **d**, **e** Cells were treated with CHX for the indicated time points and western blot analysis of Bmi1 and GAPDH was performed (**d**). The densitometric quantification of Bmi1 normalized to GAPDH was plotted against various time points to determine its half-life (**e**). **f** Cells were treated with MG132 (10 μM) or lysosomal inhibitor Chloroquine (25 μM) as indicated. Cells were subjected to western blot analysis. **g** Cells were treated with the indicated concentration of β-Asarone for 72 h and PCR analysis of p16INK4a, p14ARF and GAPDH was performed

We noticed that β-Asarone treatment significantly suppressed the expression of cancer stem cell-related proteins (c-Myc and Bmi1) in a dose-dependent manner (Fig. 2c). Among these cancer stem cell-related proteins, β-Asarone induced suppression of Bmi1 expression was more obvious than others (Fig. 2c). We speculated that Bmi1 expression was critical for the β-Asarone reduced stem-like population. Indeed, β-Asarone treatment failed to suppress the stem-like population when Bmi1 was overexpressed (Fig. 6a, b). These data implied that inhibition of Bmi1 expression was required for the β-Asarone reduced stem-like population.

### β-Asarone destabilizes Bmi1 via proteasome-related pathway

We next evaluated how β-Asarone regulated Bmi1 expression. We found that β-Asarone did not suppress Bmi1 expression at the transcription level (Fig. 6c). We thus examined whether β-Asarone might affect Bmi1 degradation. The half-life of Bmi1 was evaluated by inhibiting the de novo protein synthesis using cycloheximide treatment for different time points (0–120 min). Our data showed that Bmi1 turned over with a half-life of approximately 28.45±5.73 min (Fig. 6d). However, treatment with β-Asarone significantly accelerated Bmi1 turn over with a half-life of 10.94±2.90 min (Fig. 6d). To determine whether Bmi1 turnover involves in the lysosomal and/or proteasomal pathway, the pharmacological inhibitors of these pathways was used. Our data showed that treatment of cells with the MG132, 26S proteasomal inhibitor, but not the chloroquine, lysosomal inhibitor, recovered Bmi1 expression under β-Asarone treatment (Fig. 6f). Thus, β-Asarone promoted Bmi1 degradation via the 26S-proteasome pathway. Bmi1 is the critical component of polycomb repressive complex 1 which regulates multiple genes expression [22]. We examined whether β-Asarone might also regulate downstream target genes of polycomb repressive complex 1. Our data showed that inhibition of Bmi1 by β-Asarone treatment indeed promoted p16INK4a and p14ARF, inhibited by polycomb repressive complex 1 [22], expression (Fig. 6g).

## Discussion

In the current study, we showed that β-Asarone functioned as a sensitizer of doxorubicin and an inhibitor of the stem-like cell population in Raji lymphoma cells. We demonstrated that β-Asarone inhibited proliferation and induced apoptosis by suppressing NF-κB signaling. While the inhibitory effect of β-Asarone on stem-like cell population was caused by destabilizing Bmi1 via proteasome-related pathway.

Resistance to chemotherapy, cancer metastasis, and cancer relapse are major clinical challenges attributed to cancer stem cells. Currently, numerous therapeutic strategies have been suggested to against cancer stem cells by suppressing critical signaling pathways, regulating oncogene functions, targeting surface biomarkers, inhibiting drug-efflux pumps, enhancing immune responses and modulating the tumor microenvironment [31]. Novel compounds that selectively target cancer stem cells have been identified and evaluated in preclinical studies. For example, metformin selectively kills CD44^high^/CD24^low^ breast cancer stem cells and had a synergistic effect with doxorubicin on killing breast cancer stem cells in vitro and in vivo [32]. Salinomycin is also shown to selectively kills breast cancer stem cells [33]. Recently, natural compounds are also shown to target cancer stem cells or sensitize cancer stem cells to anti-cancer drugs. Sulforaphane, an isothiocyanate enriched in broccoli, inhibits mammosphere formation and suppresses the ALDH-positive cells of breast cancer in vitro and in vivo [34]. Moreover, quercetin, a plant-derived flavonoid found, reduces self-renewal, diminished ALDH1 activity, reverts apoptosis resistance and induces synergistic anti-cancer effects with sulforaphane [35]. In the present study, our data showed that β-Asarone, the main bioactive constituents of the traditional medical herb *Acorus calamus*, could be a novel agent for targeting the stem-like population in lymphoma cells. According to our data, β-Asarone might target the stem-like population through mechanisms related to promote Bmi1 degradation, inhibit the activity of detoxifying enzymes ALDH and increase intracellular uptake of drugs.

Polycomb group proteins have long been linked to the development of different forms of cancer [36]. Polycomb complexes are recruited to polycomb responsive elements in chromatin to epigenetic silence genes expression [37]. Bmi1 is a critical component of the polycomb complexes. Accumulating evidence has demonstrated that Bmi1 is also involved in the regulation of self-renewal, differentiation, and cancer stem cells properties. It has been reported that stem cells might bypass senescence and immortalize by cooperating Bmi1 and c-Myc signaling to downregulate p16INK4a and p19ARF expression [38]. Moreover, Bmi1 might enhance self-renewal by inducing hTERT activity to stabilize telomeres in normal mammary epithelial cells [39]. Additionally, Bmi1 could be a critical downstream target of hedgehog pathway and participate in the hedgehog pathway to modulate self-renewal of medulloblastoma brain tumor-initiating cells [40]. Besides, Bmi1 upregulates miR-21 and miR-34a by activating Akt-NF-κB pathway to regulate stem cell-like properties of gastric cancer cells [41]. In this study, our data provided evidence to show that β-Asarone might participate in the regulation of polycomb complexes related signaling by modulating protein stability of Bmi1. We observed that β-Asarone treatment decreased the expression of Bmi1 and c-Myc and increased the expression of p16INK4a, indicating that β-Asarone might also suppress stem-like properties by activating senescence and inhibiting immortalization. Although previous study shows that Bmi1 might cooperate with NF-κB to regulate stem cell-like properties [41], our data indicated that Bmi1 might regulate stem cell-like properties independent of NF-κB signaling in lymphoma cells.

Increase intracellular uptake of drugs is an attractive strategy against cancer. Tumors usually display abnormal tissue architecture and composition that limit the uptake and distribution of antitumor drugs. Numerous studies have provided various strategies to increase intracellular uptake of drugs in cancer. It has been reported that angiotensin II is utilized to induce hypertension for 15–20 min to improve the delivery of a polymeric antitumor drugs [42]. TNF-α is able to alter endothelial barrier function and tumor interstitial pressure to enhance the penetration of chemotherapeutic drug [43]. Stromal barriers reduce the penetration of chemotherapeutic drugs. Collagenase treatment significantly increases the macromolecular interstitial diffusion in penetration-resistant tumors [44]. Additionally, the combination of ABC transporters targeting agents with conventional cytotoxic drugs could lead to a potentiated effect. For example, flavonoids inhibit the function of ABC efflux transporters, mainly P-glycoprotein, MRP1, MRP2, and BCRP, to increase the effect of conventional cytotoxic drugs [45]. Moreover, depletion of cholesterol from cancer cells by methyl-β-cyclodextrin enhances doxorubicin-induced cell death by increasing intracellular doxorubicin levels [26, 46]. In the current study, we showed that β-Asarone also could be used to increase intracellular doxorubicin levels. It has been reported that Bmi1 regulates the expression of ABC transporters such as ABCG2 and ABCC1 [47]. We speculated that inhibition of Bmi1 might also important for β-Asarone induced intracellular doxorubicin uptake.

**Fig. 7:**
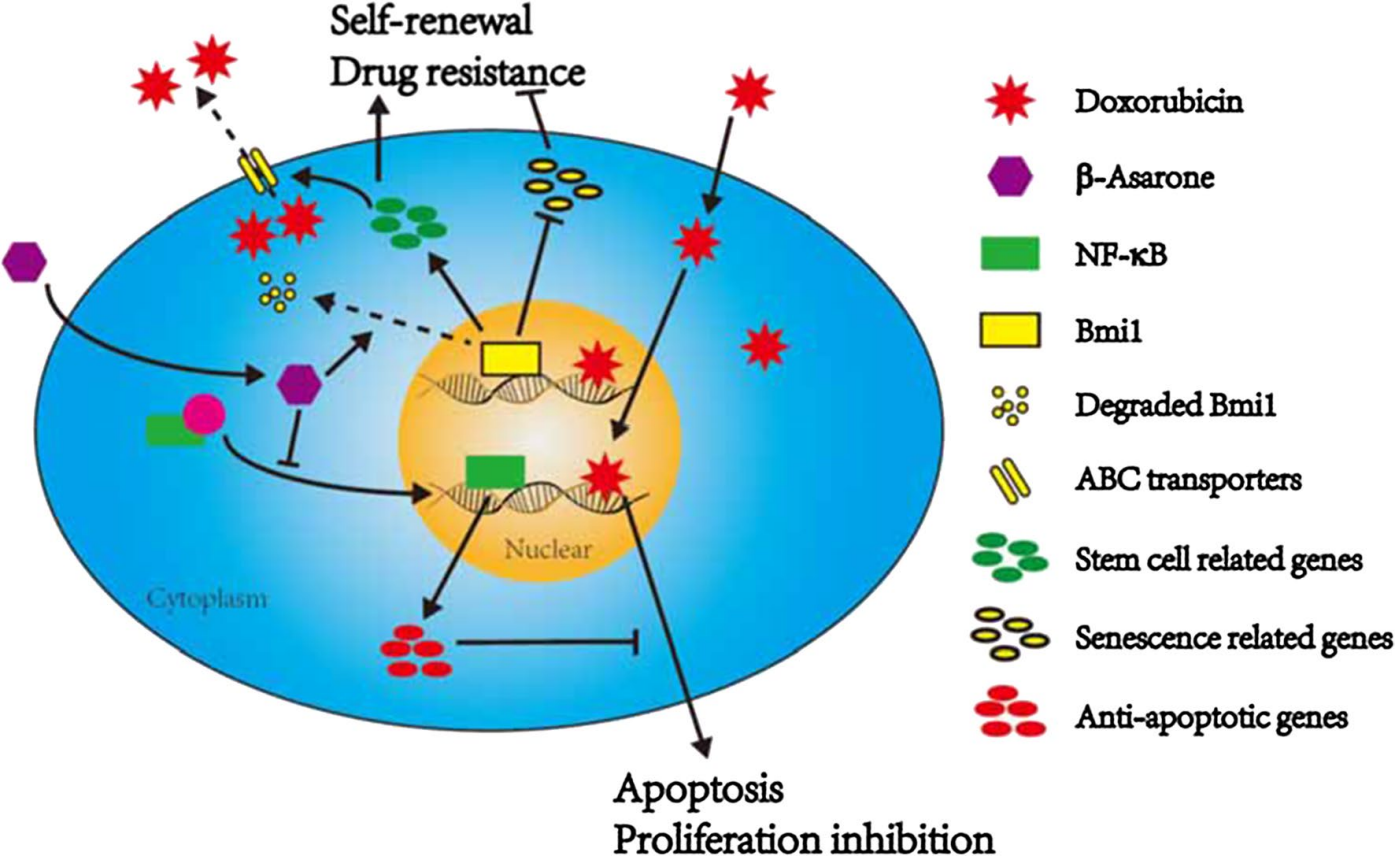
Schematic diagram showing the action of β-Asarone in the induction of apoptosis and suppression of stem-like properties in lymphoma cells. β-Asarone functioned as an inhibitor of the stem-like population in lymphoma cells by destabilizing Bmi1 via a proteasome-mediated mechanism and promoting intracellular doxorubicin uptake. β-Asarone also functioned as a sensitizer to induce synergistic effects on proliferation and apoptosis when combined with doxorubicin treatment by blocking NF-κB signaling activation

In summary, our data suggested that β-Asarone could be a potential drug for the treatment of lymphomas by increasing doxorubicin sensitivity via inhibiting NF-κB signaling and by targeting stem-like population to overcome drug resistance via reducing Bmi1 stability (Fig. 7).

## Conclusion

We revealed a novel function of β-Asarone which acted as an inhibitor of the stem-like population in lymphoma cells by destabilizing Bmi1 via a proteasome-mediated mechanism. We also showed that β-Asarone functioned as a sensitizer to induce synergistic effects on proliferation and apoptosis when combined with doxorubicin treatment by blocking NF-κB signaling activation. Our data also provided a strategy to target stem-like population, lower drug dose and increase drug sensitivity of doxorubicin.

## Additional file

**Additional file 1: Figure S1.** β-Asarone inhibits NF-κB nuclear localization and the effect of the combination of β-Asarone and JSH-23 on apoptosis. **A** Raji cells were treated with 100 μM β-Asarone for 72 h. Cells were subjected to immunofluorescence staining. Representative images were shown. Images were magnified with a 100× objective. Scale bar, 10 μm. **B** Raji cells were treated with 400 μM β-Asarone and/or 7 μM JSH-23. Apoptosis was evaluated by the Annexin V-FITC/PI staining and flow cytometry analysis. Representative results are shown in the left panel and statistical results are shown in the right panel. Bar represents mean ± SD of three independent experiments (*p < 0.05, **p < 0.01, ***p < 0.001, the ANOVA test, followed by Least Significant Difference test, were used to make statistical comparisons).

## Data Availability

Not applicable.

## Abbreviations

PCR: polymerase chain reaction
TNF-α: tumor necrosis factor α
Oct4: octamer-binding transcription factor 4
Sox2: SRY (sex determining region Y)-box 2
Klf4: Kruppel-like factor 4
PARP: poly ADP ribose polymerase
GAPDH: glyceraldehyde 3-phosphate dehydrogenase
DMSO: dimethylsulfoxide
ATCC: American Type Culture Collection
PI: propidium iodide
EGF: epidermal growth factor
bFGF: basic fibroblast growth factor
CI: combination index
RT: room temperature
ALDH: aldehyde dehydrogenases
DEAB: *N*,*N*-diethylaminobenzaldehyde
