# Supplementary figures and images for: β-Asarone increases doxorubicin sensitivity by suppressing NF-κB signaling and abolishes doxorubicin-induced enrichment of stem-like population by destabilizing Bmi1
Source: Cancer Cell Int. 2019 Jun 3;19:153. doi: 10.1186/s12935-019-0873-3 (PMC6547485; doi:10.1186/s12935-019-0873-3)

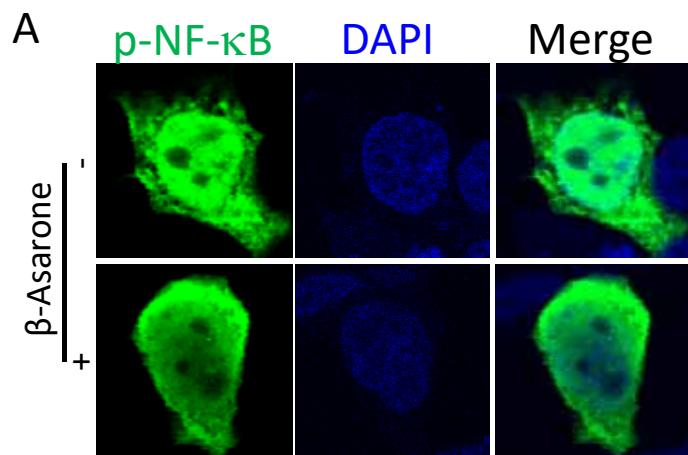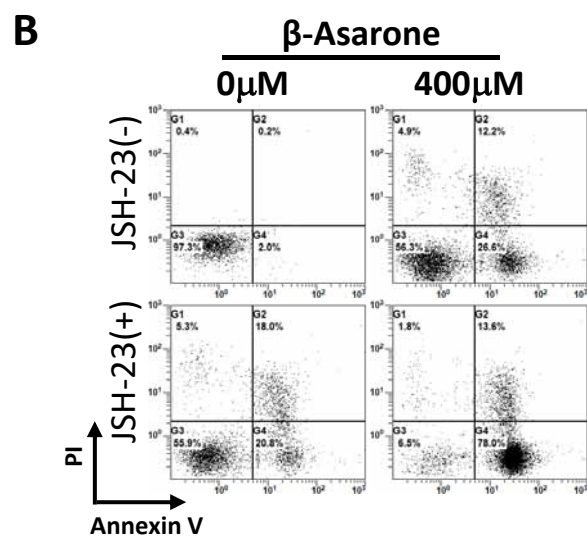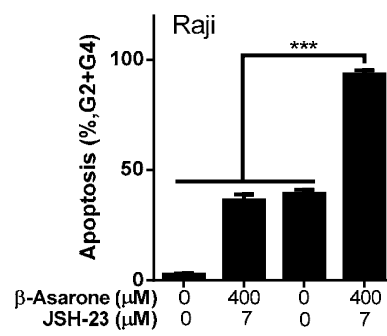

Fig. S1

Supplement: Supplementary file 1 — Additional file 1: Figure S1. β-Asarone inhibits NF-κB nuclear localization and the effect of the combination of β-Asarone and JSH-23 on apoptosis. A Raji cells were treated with 100 μM β-Asarone for 72 h. Cells were subjected to immunofluorescence staining. Representative images were shown. Images were magnified with a 100× objective. Scale bar, 10 μm. B Raji cells were treated with 400 μM β-Asarone and/or 7 μM JSH-23. Apoptosis was evaluated by the Annexin V-FITC/PI staining and flow cytometry analysis. Representative results are shown in the left panel and statistical results are shown in the right panel. Bar represents mean ± SD of three independent experiments (*p < 0.05, **p < 0.01, ***p < 0.001, the ANOVA test, followed by Least Significant Difference test, were used to make statistical comparisons). [file 12935_2019_873_MOESM1_ESM.pdf]
